# Objective Evaluation of Chronic Low-Back Pain Using Serum Lipids: The Role of the Doctor-Patient Relationship

**DOI:** 10.1155/2023/9972093

**Published:** 2023-03-30

**Authors:** Tomáš Bruthans, Jana Vránová, Anna Yamamotová

**Affiliations:** ^1^Charles University, Third Faculty of Medicine, Department of Physiology, Prague, Czech Republic; ^2^Department of Neurology, Hospital Horovice, Horovice, Czech Republic; ^3^Charles University, Third Faculty of Medicine, Department of Medical Biophysics and Medical Informatics, Prague, Czech Republic

## Abstract

Statistical data show that pain intensity in patients with low back pain is associated with a higher BMI, total serum cholesterol, and triacylglycerol levels. The objective of our study was to evaluate how these associations are dependent on the nature of the patient-doctor relationship. Eighty-nine patients hospitalized with chronic low-back pain (50 women, 39 men; average age: 64.5 ± 12.7 years) were assessed over a 3-year period. A serum lipid analysis was conducted (LDL-C, HDL-C, and total cholesterols) at admission in parallel with a subjective evaluation of pain intensity, which was assessed using a numeric rating scale. The participating physician assigned, based on their personal interaction with the patient, an attribute of affinity (positive, neutral, and negative) towards them. Current serum lipid levels and pain intensity were correlated relative to these attributes. Pain intensity did not differ between the groups assigned positive or negative attributes of affinity. In patients belonging to the “positive” group, pain intensity correlated positively with total cholesterol (*p*=0.01) and LDL cholesterol (*p*=0.007). No correlations were found in the “negative” group or when the patient-doctor relationship was ignored. We found a significant association between subjectively assessed low back pain intensity and serum levels of total and LDL cholesterol in patients with whom the physician had a positive affinity. A positive affinity with the patients having chronic pain and the patient's trust in their physicians may ultimately mean that the patient's statement about pain is more credible, which may retroactively affect the outcome of therapy.

## 1. Introduction

Chronic pain is an indirectly observable phenomenon. There are no quick and accurate objective methods for evaluating its intensity. Objective electrophysiological methods, neuroimaging, and mapping the activity of the autonomic nervous system have all been used to assess acute as well as chronic pain, but they are not practical for use in everyday practice [1–5]. In common clinical practice, questionnaires are often used to assess chronic pain, which is reproducible and easy to administer [6, 7]. The McGill University Questionnaire is a commonly used self-assessment questionnaire, where patients provide their physicians with subjective information about the intensity and quality of the pain they experience [7, 8]. The Brief Pain Inventory (BPI) and the clinically popular short-form BPI-SF are other common methods which assess, in addition to the pain intensity, the location of pain indicated (on a drawing or image of a human figure), affective descriptors of pain, and medications used for pain treatment [9]. The simplest and the most commonly used assessment is the numeric rating scale (NRS-11) or the visual analog scale (VAS), where pain intensity is visually marked on a line with two extremes, no pain and maximum possible pain [10]. Both methods involve some simplification of the actually experienced pain, but the simplicity makes them easy to understand.

Previous animal and human studies have shown that both acute and chronic pain alter serum lipid levels [11–15]. The relationship between obesity and chronic vertebrogenic pain has been shown, and analyses on representative sets of patients also indicated that patients with high BMIs also had elevated cholesterol and triacylglycerol levels [16, 17].

Because blood chemistry is a routine, reproducible, and particularly easy evaluation, the goal of our work was to study the association between subjectively experienced pain and serum lipid levels in patients with chronic vertebrogenic pain but without significant obesity.

A patient's assessment of their pain, in the presence of an attending physician, can be significantly influenced by their relationship with the physician. We also have the patient's motivation to be treated, which can simulate or dissimulate pain, which can be affected by their relationship with the doctor. Empathy is the ability to share the affective states of others, or the ability to understand the pain of others, and is important in the formation of a positive doctor-patient relationship [18]. It is also associated with patient satisfaction and compliance with recommended treatments [19]. In good doctor-patient relationships, pain self-assessments by patients are considered more accurate and reliable by the physician and thus contribute to the choosing of the best therapy; it may also better reflect objectively measured clinical values.

We tested the hypothesis that patients with more intensive vertebrogenic pain would have higher plasma lipid levels, especially total cholesterol, and LDL cholesterol. Additionally, we looked at how this association could be influenced by the patient-doctor relationship, also called therapeutic alliance [20]; an association that has not been previously studied.

## 2. Methods

### 2.1. Clinical Subjects

This was a multiyear observational study. Over three years, 89 patients (39 men, 50 women) with an M51-4 or G54.2-4 diagnosis, based on the 10th ICD-revision and chronic vertebrogenic pain were evaluated at the inpatient unit of the Department of Neurology. The vertebrogenic symptom complex includes local and referred pain and autonomic reflex dysfunction within the lumbosacral zones of the head [21]. The pain can have many qualities and a number of causes as well as just as many treatment options; in addition to pharmacological treatment, it may also include physical therapy or spinal manipulation and, in justified cases, surgical treatment.

Study exclusion criteria included the following: dependence on tobacco, alcohol, or narcotics, hormonal therapy treatment, medical treatment for an endocrine disease, and psychiatric diagnosis. All patients were taking nonsteroidal anti-inflammatory drugs, 20 patients were taking antidepressants, and 32 patients were taking statins. All patients signed informed consent, and the study was approved by the local Ethics Committee.

### 2.2. Assessment

On the day of admission, blood samples were drawn from fasting patients. Serum lipids, i.e., total cholesterol (TC), LDL-cholesterol, HDL-cholesterol, and triacylglycerols (TG), were measured using an automatic biochemical analyzer in a certified laboratory. In the presence of a physician, the patients assessed their pain intensity using a numeric rating scale (NRS-11) by assigning numbers 0–10 to their pain level, where 0 was a pain-free state, and 10 was the maximum imaginable pain intensity.

At this same time, the attending physician assigned an attribute of affinity (or fellowship) based on their experience with the patient, where “+” means a high degree of affinity with the patient, “0” a neutral affinity, and “−” a negative affinity with the patient. The attitude towards the patient was based primarily on the subjective experience of mutual verbal and nonverbal communication.

### 2.3. Statistical Analyses

All quantitative variables are given as means and standard deviations, and qualitative variables were given as frequencies and percentages. These parameters were calculated both for all patients together and for two groups of patients, which were created according to the patient-doctor relationship. In the first group were the patients with positive affinity attribute and in the second group were the patients with negative or neutral attribute.

To find the relationship between pain intensity NRS-11 and serum lipids, i.e., TC, LDL-C, HDL-C, TG, and LDL/HDL ratio, as well as between NRS-11 and age and BMI of the patients, the parametric Pearson's correlation coefficients were calculated. The required sample size for all significant correlations was calculated.

After merging the indifferent and negative groups of patients, we obtained two groups of patients with positive and negative rapport. For the comparison of age, BMI, NRS-11, and all observed serum lipids between these two groups of patients, the parametric Student's *t*-test was used. To compare the qualitative variables, i.e., sex (M/F) and medication with statins (Yes/No) and antidepressants (Yes/No), the analysis of contingency tables was used; Pearson's chi-square test was calculated.

In order to evaluate the effect of all observed variables in the assessment of the severity of chronic pain (NRS-11), the general multivariable regression analysis was performed. We created three regression models—for all patients together and for each group of patients—with positive and negative rapport separately.

In order to find whether our observed variables discriminate well enough between the two groups of patients (positive vs. negative rapport), the multivariate logistic regression model was performed. The list for predictive factors was the same as in the multivariate regression model except for the addition of NRS-11.

TIBCO Statistica version 14.0 and IBM SPSS Statistics version 23 were used for statistical analysis. A *p* value less than 0.05 was considered to be statistically significant.

## 3. Results

### 3.1. Gender Differences

The mean pain intensity was the same in men and women (5.62 vs. 5.66, respectively; *p*=0.92). Men had higher TG compared to women (1.39 vs. 1.13 mmol/l, respectively; *p*=0.04), but they did not differ in levels of TC, LDL-C, and HDL-C.

### 3.2. Effect of Statins and Antidepressants

Thirty-two patients (21 men and 11 women) were taking statins for lipid-lowering therapy. The statin group had lower TC and LDL-C in comparison with untreated patients (TC: 4.86 vs. 5.42 mmol/l, respectively, *p*=0.025; LDL-C: 2.7 vs. 3.34 mmol/l, respectively, *p*=0.003). The groups did not differ relative to pain intensity.

Twenty patients (6 men and 14 women) were taking antidepressants. Neither pain intensity nor lipid levels differed from patients not taking antidepressants. The lower weight and BMI in this group reflects the greater number of women in the group.

### 3.3. Patient-Doctor Relationships (Table 1)

Forty-three patients (18 men and 25 women) received a positive affinity attribute, thirty-six patients (18 men and 18 women) received a neutral attribute, and ten patients (3 men and 7 women) received a negative attribute. Due to the small number of subjects in the last category, these patients were merged with the neutral group, creating a group that contained 21 men and 25 women.

Although positive and neutral or negative patients did not differ significantly in pain intensity, BMI, TC, or HDL-C, they did differ significantly in the LDL-C which was lower in positive affinity patients (2.86 vs. 3.34 mmol/l, respectively, *p*=0.028) and the LDL/HDL ratio which was also significantly lower in the positive affinity patients (1.82 vs. 2.27, respectively, *p*=0.004). According to the results of contingency tables analysis (Table 1 lower part), we found no statistically significant difference in distributions of men and women, usage of statins (Yes/No), and antidepressants (Yes/No) between positive and negative groups of patients.

### 3.4. Associations between Pain Intensity and Plasma Lipids (Table 2)

**Figure 1:**
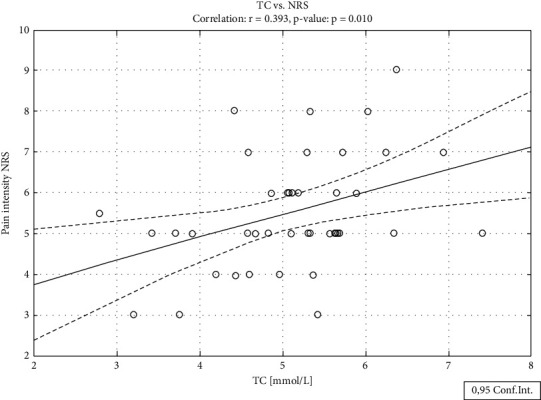
Relationship between pain intensity and total cholesterol levels in patients with whom the treating physician has a positive relationship.

**Figure 2:**
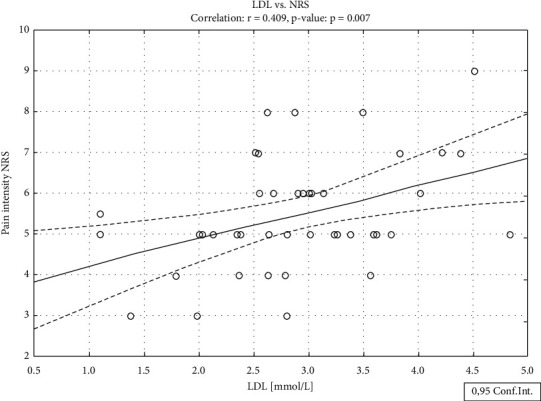
Relationship between pain intensity and LDL cholesterol levels in patients with whom the treating physician has a positive relationship.

Analyzing the relationships between pain intensity and lipid levels in the entire sample of patients, it was shown that pain did not correlate with any particular variable. In contrast, we found a significant positive correlation between TC and pain intensity (*r* = 0.393, *p*=0.010) (Figure 1), LDL-C and pain intensity (*r* = 0.409, *p*=0.007) (Figure 2), and LDL/HDL ration and pain intensity (*r* = 0.348, *p*=0.024) in the group of positive affinity patients only. Required sample size conditions were almost fulfilled only for TC (required sample size 48) and LDL-C (required sample size 45). Neither HDL nor TG nor age and nor BMI correlated with pain intensity in any group.

### 3.5. General Regression Model

In order to evaluate the effect of serum lipids, age, BMI, sex, and medication with statins and antidepressants on the assessment of the severity of chronic pain, general multivariable regression analysis was performed. As the independent variable, we used all serum lipids, i.e., TC, LDL-C, HDL-C, TG, and LDL/HDL ratio, and then age, BMI, sex, and medication with statins and antidepressants; as the dependent variable, the pain intensity was used. We created three regression models—for all patients together and for each group of patients—with positive and negative rapport separately. All three models were statistically insignificant, and neither model was significant for predicting the severity of chronic pain, NRS-11.

The values of coefficients of determination *R*^2^ are as follows: positive rapport group: *R*^2^ = 0.249, negative rapport group: *R*^2^ = 0.169, and the entire sample of patients, as the input factor, the rapport between the patient and the doctor (positive vs. negative) was added. The coefficient of determination *R*^2^ = 0.103, the only variable for which the *p* value is less than 0.1, was the LDL/HDL ratio (*p*=0.087).

### 3.6. Logistic Regression Model

In order to find whether our observed variables discriminate well enough between the two groups of patients (positive vs. negative rapport), the multivariate logistic regression model was performed. The list for predictive factors was the same as in the multivariate regression model except for the addition of NRS-11.

The results are as follows: 2LL statistics is insignificant if *p*=0.352, Nagelkerke *R*^2^ is only 0.146, and for the Hosmer–Lemeshow test, the *p* value is 0.490; the model adequately interpolated the data. The classification ability is only 59.3%. The area under the ROC curve (AUC) which determines the discrimination power of the logistic model reached the value 0.695; discrimination quality according to Tape [22] is “Poor.” No input variable is statistically significant.

## 4. Discussion

### 4.1. Pain and Serum Lipids

In our study, we found a positive correlation between the subjectively evaluated intensity of chronic vertebrogenic pain and serum total and LDL cholesterol levels in adult patients with whom the attending physician had a relationship describable as positive affinity.

Experiments with animals have shown that serum lipids might reflect a nonspecific stress effect of acute and chronic pain [11]. After acute painful stimulation, HDL-cholesterol, triacylglycerols, glucose, and free fatty acids were elevated, whereas total cholesterol levels did not change and long-term repeated painful stimulation resulted in an increase in LDL-cholesterol and HDL-cholesterol. The question is, to what extent this relationship also applies to humans.

Higher serum levels of triglycerides and HDL-cholesterol were detected in the acute pain of patients with fractures and acute pancreatitis [12]. These values subsequently decreased during hospitalization and treatment. Even in hospitalized pain-free controls, higher levels of LDL-cholesterol and triacylglycerols have been reported, which is believed to be the effect of immobilization stress during hospitalization.

In several studies, atherogenic lipids, LDL cholesterol, and triglycerides were associated with clinical manifestations of lumbalgia (low back pain) to support the lumbar atherosclerosis hypothesis as a cause of intervertebral disc degeneration or chronic hip pain [23, 24]. On the other hand, the recent studies have questioned this hypothesis and consider it insufficiently substantiated [25].

Conversely, there is a work showing a decrease in total cholesterol in acute severe trauma. Patients with good outcomes in intensive care units showed improvement in TC levels during treatment, while a further reduction was observed in patients with infections, organ dysfunction, or death [26].

It is also important to note that each disease has its own dynamics. During illnesses, lipid levels may change and different active substances, not only lipids, may differently affect pathological pain processes during the transition from acute to chronic pain [15].

### 4.2. Gender Differences

Compared to women, men have higher triglycerides and total cholesterol levels and lower HDL-C-levels [27]. Also, total- and LDL-cholesterol appears to be more important in determining cardiovascular diseases in men, while high triacylglycerols and low HDL-cholesterol are more significant in women [28].

In men, the total cholesterol and triacylglycerol levels correlated with the five-year probability of developing lumbar vertebrogenic algic syndrome. This relationship has not been demonstrated in women [29]. Another study describes an inverse association between the prevalence of low back pain with HDL-C and a positive association with triacylglycerols, with stronger associations in women than in men [24, 30]. A combination of higher total- and LDL-cholesterol levels has also been reported in patients with myofascial pain [31].

### 4.3. Statins and Antidepressants

The role of statins in influencing pain is not yet fully clear. Attenuation of thermal hyperalgesia in an animal model of neuropathic pain, induced by partial ligation of the sciatic nerve, was independent on the statin-induced hypolipidemic effect [32].

In our study, patients taking statins had lower TC and LDL-C in comparison with patients without this treatment. Contrary to the expectation, triglycerides in these patients were increased. This observation can be explained by statins being effective at decreasing triglyceride levels but only in hyper-triglyceridemic patients [33].

Even though statins also exert a pleiotropic nonlipid effect, they possess anti-inflammatory properties and antioxidant and neuromodulatory effects; we did not observe any effect on pain intensity in patients treated with statins [34]. The controversial results following from animal and clinical studies did not permit simple conclusions about whether statins have a pain-inducing or pain-attenuating role [35].

Several studies have demonstrated frequent co-occurrences of pain and depression. Chronic pain is associated with changes in brain physiology and anatomy, and the positive impact of antidepressants might result in a reduction of these pathological processes and in the amelioration of symptoms, which can improve the quality of the life of patients [13, 36]. Therefore, the antidepressant use was another analyzed factor. Although we did not evaluate the presence of clinical depression, almost one-fourth of our patients were taking antidepressants as adjuvant therapy. We found no differences either in pain or in other serum lipid biomarkers between these two groups of patients. The lower weight and BMI of the patients taking antidepressants can be explained by the greater occurrence of women in this group.

### 4.4. Affinity

The most important result of our study was finding a positive correlation between the subjectively evaluated intensity of chronic vertebrogenic pain and serum total and LDL cholesterol levels in adult patients with whom the attending physician had a relationship describable as a positive affinity.

Here is the distinction between affinity, which can be understood as an affirmative connection with the feelings of another person and empathy, which represents an act of understanding without the need to agree with the feelings of another [37]. Empathy as a psychological phenomenon can be investigated using questionnaires and thus offers the potential to objectify certain physiological relationships in the future [38]. The attending physician in our study (T.B.) was a neurologist, and according to a comparative study conducted on a group of healthcare professions, he is in the category of specialists that have higher levels of empathy, as evaluated using the Jefferson scale of empathy [37].

The positive correlation between pain and serum lipids is certainly influenced by the patient's own assessment of pain. This means that the self-assessment of pain intensity may be somewhat affected by a therapeutic relationship in which it can function as a placebo or nocebo effect. According to Fabrizio Benedetti, meeting the doctor involves many psychological responses in the patient's brain, which are responsible things such as expectations, trust, and hope. Similarly, many mechanisms are at work in a doctor's brain, such as empathy and compassion. In turn, these led to the final step of providing therapy, which regardless of its effectiveness or ineffectiveness, triggers a placebo response” [39].

### 4.5. Limitation of the Study

Many studies have shown that physical activity is a safe method for improving patients' physical performance and alleviating symptoms. Both high-intensity aerobic exercise and long-term low-intensity exercise were found to reduce pain, the disability rate, and psychological stress and enhance quality of life in patients with low back pain [40, 41]. In our study, the physical activity of the monitored patients was not controlled.

The impact of exercise on the lipid profile is somewhat controversial; a number of data confirm the beneficial effects of the regular activity on cholesterol levels; however, significant inconsistency in the blood lipid response has been observed. The most frequently observed change was an increase in HDL-C with less frequently observed decrease in TC, LDL-C, and TG [42, 43].

We consider the quality of the therapeutic relationship to be crucial for an objective verbal assessment of pain. Although the number of patients in our work is limited and the verification of the relationship presented in this study deserve more extensive research in relation to gender, age, and cultural habits, we consider a good therapeutic doctor-patient relationship a good starting point for more objective pain assessment.

We would like to believe the research results will be similar even in selecting and assessing other patients by another therapist. Rapport is not a personality trait, and individuals experience rapport as the result of a combination of qualities that emerge from each individual during interaction [21]. This hypothesis should be the aim of further research.

In conclusion, we found a positive correlation between subjectively evaluated chronic vertebrogenic pain intensity and serum total and LDL cholesterol levels in adult patients treated by a physician who described their relationship as having the attribute of positive affinity. This positive relationship between a physician and a patient with chronic pain, as well as the patient's confidence in the physician, may result in a more credible pain assessment by the patient, which may retroactively affect the outcome of the therapy.

## Figures and Tables

**Table 1:** Main characteristics of the patients.

	All patients (*N* = 89)	Rapport		*p* value of Student's *t*-test
		Positive (*N* = 43)	Negative (*N* = 46)	
	Mean ± SD	Mean ± SD	Mean ± SD	
Age (years)	63.42 ± 12.69	64.51 ± 13.79	62.39 ± 11.63	0.434
BMI (kg·m^−2^)	28.17 ± 4.29	27.54 ± 4.24	28.77 ± 4.30	0.186
Pain (NRS-11)	5.65 ± 1.48	5.48 ± 1.36	5.80 ± 1.57	0.300
TC (mmol/l)	5.22 ± 1.13	5.03 ± 0.96	5.40 ± 1.25	0.126
LDL-C (mmol/l)	3.11 ± 1.01	2.86 ± 0.87	3.34 ± 1.08	0.028^*∗*^
HDL-C (mmol/l)	1.56 ± 0.32	1.63 ± 0.30	1.51 ± 0.33	0.079
TG (mmol/l)	1.24 ± 0.60	1.22 ± 0.64	1.27 ± 0.56	0.694
LDL/HDL	2.04 ± 0.74	1.82 ± 0.65	2.27 ± 0.76	**0.004**

Qualitative variable	*N* (%)	*N* (%)	*N* (%)	*p* value of Pearson's chi-square test
Sex				
Male	39 (43.82)	18 (41.86)	21 (45.65)	0.719
Female	50 (56.18)	25 (58.14)	25 (54.35)	
Statins				
Yes	32 (35.96)	17 (39.53)	15 (32.61)	0.496
No	57 (64.04)	26 (60.47)	31 (67.39)	
Antidepressants				
Yes	20 (22.47)	10 (23.26)	10 (21.74)	0.864
No	69 (77.52)	33 (76.74)	36 (78.26)	

^*∗*^Statistically significant values are marked in bold. Means and standard deviations (SD) for quantitative variables and numbers and percentages of qualitative variables for all patients together (*N* = 89) and for two groups of patients (positive (*N* = 43) and negative (46) rapport). *p* values of Student's *t*-test and Pearson's chi-square test were used to compare positive and negative groups.

**Table 2:** Parametric Pearson's correlation coefficients, *p*-values, and required sample sizes (only for statistically significant coefficients).

Pain (NRS) versus	Age	BMI	TC	LDL-C	HDL-C	TG	LDL/HDL
*All patients (N* *=* *89)*							
Correlation coefficient *r*	−0.133	0.073	0.153	0.143	0.067	0.020	0.045
*p* value	0.223	0.505	0.160	0.189	0.540	0.861	0.681

*Rapport: positive (N* *=* *43)*							
Correlation coefficient *r*	−0.090	0.140	0.393	0.409	−0.006	0.085	0.348
*p* value	0.570	0.376	0.010^*∗*^	**0.007**	0.972	0.594	**0.024**
Required sample size^†^			48 (64^‡^)	45 (59^‡^)			63 (83^‡^)

*Rapport: negative (N* *=* *46)*							
Correlation coefficient *r*	−0.171	−0.007	−0.018	−0.059	0.154	−0.052	−0.208
*p* value	0.268	0.967	0.908	0.702	0.317	0.738	0.175

^*∗*^Statistically significant values are marked in bold. ^†^Required sample size is calculated only for statistically significant correlation coefficients (given parameters for required sample size calculation are as follows: *α* = 0.05, *β* = 0.2). ^‡^Required sample size for *α* = 0.05 and *β* = 0.1.

## Data Availability

The data set used to support the findings of this study is available from the corresponding author (yamamoto@lf3.cuni.cz) upon request.

## References

[1] Handwerker H. O., Kobal G. (1993). Psychophysiology of experimentally induced pain. *Physiological Reviews*.

[2] Lee M. C., Tracey I. (2013). Imaging pain: a potent means for investigating pain mechanisms in patients. *British Journal of Anaesthesia*.

[3] Garcia J. B. S., Garcia J. B. S., Viera E. B., Santos A. M., Bertrand R. H. (2013). Pupillometry: the influence of gender and anxiety on the pain response. *Pain Physician*.

[4] Ellermeier W., Westphal W. (1995). Gender differences in pain ratings and pupil reactions to painful pressure stimuli. *Pain*.

[5] de Tommaso M., Ricci K., Libro G. (2017). Pain processing and vegetative dysfunction in fibromyalgia: a study by sympathetic skin response and laser evoked potentials. *Pain Res Treat*.

[6] Melzack R., Melzack R. (1983). The McGill pain questionnaire. *Pain Measurement and Assessment*.

[7] Noble B., Clark D., Meldrum M. (2005). The measurement of pain, 1945-2000. *Journal of Pain and Symptom Management*.

[8] Melzack R., Torgerson W. S. (1971). On the language of pain. *Anesthesiology*.

[9] Cleeland C. S., Osoba D. (1991). Pain assessment in cancer. *Effect of Cancer on Quality of Life*.

[10] Dworkin R. H., Turk D. C., Farrar J. T. (2005). Core outcome measures for chronic pain clinical trials: IMMPACT recommendations. *Pain*.

[11] Rokyta R., Kleppinger J. (1998). Biochemical and electrophysiological changes in animal models of chronic and visceral pain. *Pain Clinical Aspects and Therapeutical Issues, Part III. Linz*.

[12] Křikava K., Kalla K., Yamamotová A., Rokyta R. (2004). Blood serum changes in patients with pain during bone fractures and acute pancreatitis. *Neuroendocrinology Letters*.

[13] Rokyta R., Haklova O., Yamamotova A. (2009). Assessment of chronic benign and cancer pain using blood plasma biomarkers. *Neuroendocrinology Letters*.

[14] Yamamotová A., Srámková T., Rokyta R. (2010). Intensity of pain and biochemical changes in blood plasma in spinal cord trauma. *Spinal Cord*.

[15] Rokyta R., Yamamotová A., Sulc R., Trefil L., Racek J., Treska V. (2008). Assessment of biochemical markers in patients with pain of vascular origin. *Clinical and Experimental Medicine*.

[16] Epstein R. M., Hadee T., Carroll J., Meldrum S. C., Lardner J., Shields C. G. (2007). „Could this be something,?“ Reassurance, uncertainty, and empathy in response to patients´ expressions of worry. *Journal of General Internal Medicine*.

[17] Wilsgaard T., Arnesen E. (2004). Change in serum lipids and body mass index by age, sex, and smoking status: the Tromsø study 1986-1995. *Annals of Epidemiology*.

[18] Singer T., Seymour B., O’Doherty J., Kaube H., Dolan R. J., Frith C. D. (2004). Empathy for pain involves the affective but not sensory components of pain. *Science*.

[19] Shiri R., Karppinen J., Leino-Arjas P., Solovieva S., Viikari-Juntura E. (2010). The association between obesity and low back pain: a meta-analysis. *American Journal of Epidemiology*.

[20] Leach M. J. (2005). Rapport: a key to treatment success. *Complementary Therapies in Clinical Practice*.

[21] Jinkins J. R., Whittemore A. R., Bradley W. G. (1989). The anatomic basis of vertebrogenic pain and the autonomic syndrome associated with lumbar disk extrusion. *American Journal of Roentgenology*.

[22] Tape T. G. (2020). The area under an ROC curve [online], university of Nebraska medical center. *Interpreting Diagnostic Tests, Nebraska Medical Center*.

[23] Leino-Arjas P., Kauppila L., Kaila-Kangas L., Shiri R., Heistaro S., Heliovaara M. (2008). Serum lipids in relation to sciatica among Finns. *Atherosclerosis*.

[24] Heuch I., Heuch I., Hagen K., Zwart J.-A. (2014). Do abnormal serum lipid levels increase the risk of chronic low back pain? The Nord-Trøndelag Health Study. *PLoS One*.

[25] Shcherbina A., Longacre M. (2017). The association between atherosclerosis and low back pain: a systematic review. *PM&R*.

[26] Dunham C., Fealk M. H., Sever W. E. (2003). Following severe injury, hypocholesterolemia improves with convalescence but persists with organ failure or onset of infection. *Critical Care*.

[27] Seidell J. C., Cigolini M., Charzewska J. (1991). Fat distribution and gender differences in serum lipids in men and women from four European communities. *Atherosclerosis*.

[28] Vitale C., Fini M., Speziale G., Chierchia S. (2010). Gender differences in the cardiovascular effects of sex hormones. *Fundamental & Clinical Pharmacology*.

[29] Leino-Arjas P., Kaila-Kangas L., Solovieva S., Riihimäki H., Kirjonen J., Reunanen A. (2006). Serum lipids and low back pain: an Association? A follow-up study of a working population sample. *Spine*.

[30] Heuch I., Heuch I., Hagen K., Zwart J.-A. (2010). Associations between serum lipid levels and chronic low back pain. *Epidemiology*.

[31] Ozgocmen S., Ardicoglu O. (2000). Lipid profile in patients with fibromyalgia and myofascial pain syndromes. *Yonsei Medical Journal*.

[32] Kamel E. M., Elsaid A. F., Gumaa E. A., El Sheweal A. E. M. (2016). Statins attenuate hyperalgesia and inflammation in experimentally induced acute and chronic pain in rats. *Ain-Shams Journal of Anaesthesiology*.

[33] Stein E. A., Lane M., Laskarzewski P. (1998). Comparison of statins in hypertriglyceridemia. *The American Journal of Cardiology*.

[34] Wierzbicki A. S., Poston R., Ferro A. (2003). The lipid and non-lipid effects of statins. *Pharmacology & Therapeutics*.

[35] Bhalla S., Singh N., Jaggi A. S. (2014). Statins: do they aggravate or ameliorate neuropathic pain?. *The Journal of Pain*.

[36] Nekovarova T., Yamamotova A., Vales K., Stuchlik A., Fricova J., Rokyta R. (2014). Common mechanisms of pain and depression: are antidepressants also analgesics?. *Frontiers in Behavioral Neuroscience*.

[37] Hojat M., Gonnella J. S., Mangione S., Nasca T. J., Magee M. (2003). Physician empathy in medical education and practice: experience with the Jefferson scale of physician empathy. *Seminars in Integrative Medicine*.

[38] Marci C. D., Ham J., Moran E., Orr S. P. (2007). Physiologic correlates of perceived therapist empathy and social-emotional process during psychotherapy. *The Journal of Nervous and Mental Disease*.

[39] Benedetti F. (2013). Placebo and the new physiology of the doctor-patient relationship. *Physiological Reviews*.

[40] Gordon R., Bloxham S. (2016). A systematic review of the effects of exercise and physical activity on non-specific chronic low back pain. *Health Care*.

[41] Luan X., Tian X., Zhang H. (2019). Exercise as a prescription for patients with various diseases. *Journal of Sport and Health Science*.

[42] Mann S., Beedie C., Jimenez A. (2014). Differential effects of aerobic exercise, resistance training and combined exercise modalities on cholesterol and the lipid profile: review, synthesis and recommendations. *Sports Medicine*.

[43] Leon A. S., Sanchez O. A. (2018). Response of blood lipids and lipoproteins to exercise training alone or combined with dietary intervention. *Medicine & Science in Sports & Exercise*.

